# The Accuracy of a Screening System for Carpal Tunnel Syndrome Using Hand Drawing

**DOI:** 10.3390/jcm10194437

**Published:** 2021-09-27

**Authors:** Takuro Watanabe, Takafumi Koyama, Eriku Yamada, Akimoto Nimura, Koji Fujita, Yuta Sugiura

**Affiliations:** 1 School of Science for Open and Environmental Systems, Graduate School of Science and Technology, Keio University, Kanagawa 223-8522, Japan; t-tawatana@keio.jp (T.W.); sugiura@keio.jp (Y.S.); 2 Department of Orthopedic and Spinal Surgery, Graduate School of Medical and Dental Sciences, Tokyo Medical and Dental University, Tokyo 113-8510, Japan; koya.orth@tmd.ac.jp (T.K.); erikorth@tmd.ac.jp (E.Y.); 3 Department of Functional Joint Anatomy, Graduate School of Medical and Dental Sciences, Tokyo Medical and Dental University, Tokyo 113-8510, Japan; nimura.orj@tmd.ac.jp

**Keywords:** carpal tunnel syndrome, support vector machine, machine learning, tablet app, screening, manual dexterity, drawing, nerve, pain, mobility

## Abstract

When carpal tunnel syndrome (CTS), an entrapment neuropathy, becomes severe, thumb motion is reduced, which affects manual dexterity, such as causing difficulties in writing; therefore, early detection of CTS by screening is desirable. To develop a screening method for CTS, we developed a tablet app to measure the stylus trajectory and pressure of the stylus tip when drawing a spiral on a tablet screen using a stylus and, subsequently, used these data as training data to predict the classification of participants as non-CTS or CTS patients using a support vector machine. We recruited 33 patients with CTS and 31 healthy volunteers for this study. From our results, non-CTS and CTS were classified by our screening method with 82% sensitivity and 71% specificity. Our CTS screening method can facilitate the screening for potential patients with CTS and provide a quantitative assessment of CTS.

## 1. Introduction

Carpal tunnel syndrome (CTS) is one of the most common entrapment neuropathies [1]. The initial symptoms are numbness and sensory disturbance from the thumb to the ring finger; however, because these subjective symptoms do not significantly disrupt their daily activities, the patients do not seek medical attention [2]. As the severity of CTS increases, there is a possible failure of thumb motion, and the patients undergo carpal tunnel release surgery [3], which increases their physical burden. Furthermore, the postoperative outcome is poor and in some severe cases [4]. Conservative treatments of CTS, such as oral medications, injections, and wrist support, are expected to be effective if initiated at the early stage of CTS; therefore, early detection of CTS by screening is desirable.

Accordingly, mobile apps [5,6] have been developed to serve as a simple and objective screening tool for CTS. These apps have focused on the failure of thumb motion caused by atrophy of the thenar muscle, which affects manual dexterity, causing patients to be aware of associated symptoms, such as difficulties in fastening their buttons and writing [7]. In particular, writing plays an important role in our daily lives; therefore, it is significantly impacted by the failure of the thumb motion.

Recent advances in sensing technology have proposed pens and motion capture tools that can measure writing motion [8,9]. However, previous studies could not set up an experimental environment to measure writing motion because of the special devices and systems required. Thus, there have been attempts to evaluate writing motion using off-the-shelf tablets and styluses [10]. Since tablets can measure handwriting pressure and altitude–azimuth of styluses, tablet apps have been developed to evaluate writing motion in dysgraphia [11] and Parkinson’s disease [12]. Moreover, the use of off-the-shelf devices not only reduces the cost of preparing special experimental environments, but also reduces medical costs as a result of the early detection of diseases outside of medical institutions, such as at home [13,14,15,16].

**Figure 1:**
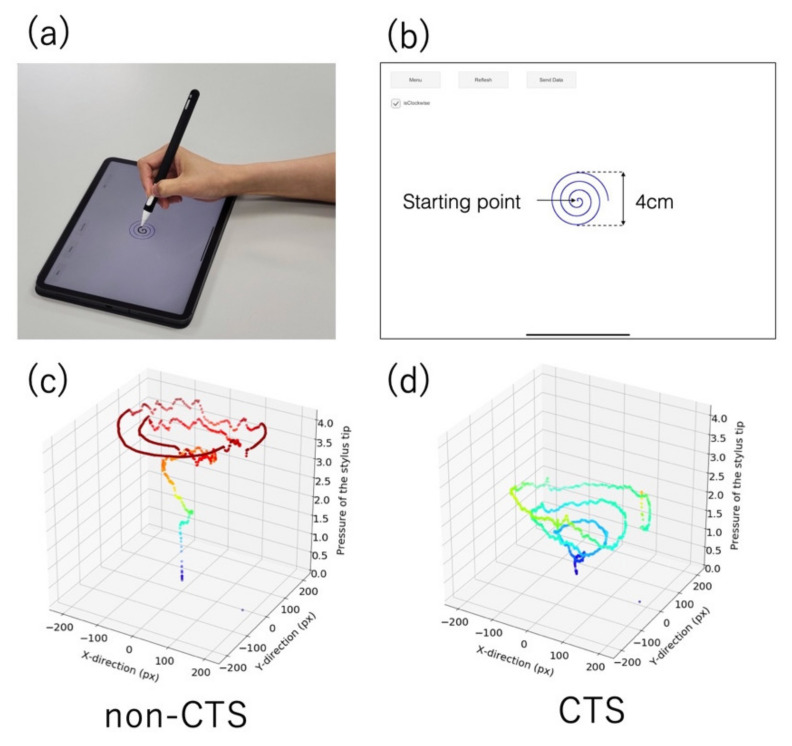
(**a**) A participant using the stylus on the app installed on the tablet. (**b**) The app screen. Participants drew a spiral along the blue guide spiral. The longitudinal diameter of the guide spiral was set to 4 cm to observe their manual dexterity. (**c**,**d**) are 3D graphs with the stylus trajectory on the xy-plane and the pressure of the stylus tip on the vertical axis drawn by non-CTS and CTS participants, respectively.

We hypothesized that sensory disturbance and the lack of manual dexterity, resulting from CTS, affect stylus manipulation, and we developed a screening method for CTS, focusing on drawing motion, using a tablet and stylus and verified for its accuracy (Figure 1a).

## 2. Materials and Methods

This study was approved by the Institutional Review Board of Tokyo Medical and Dental University. Informed consent was obtained from all the participants.

### 2.1. Participants

We recruited 33 patients with CTS and 31 healthy volunteers from July 2020 to March 2021. The CTS group was diagnosed with CTS by hand surgeons in the orthopedic outpatient clinic based on physical examinations and nerve conduction studies. The nerve conduction study, including motor and sensory nerve conduction velocity studies, was performed using Neuropack X1 (Nihon Kohden, Tokyo, Japan). The results were used to classify the severity of CTS based on the Bland classification [17]. The patients with CTS answered the Disabilities of the Arm, Shoulder, and Hand (DASH) questionnaire [18]. The score for question 2 on writing was extracted from the DASH score and recorded as the DASH score (writing) on a scale of 1–5, with 5 indicating the highest degree of writing disability. In addition, their pulp pinch strength and grip strength were measured. The non-CTS group comprised patients who had visited the orthopedic outpatient clinic or had been hospitalized for conditions other than CTS and had no hand complaints or abnormal findings upon examination by the hand surgeons. Participants with a history of upper extremity disease, injury, or surgery; those with inflammatory diseases such as rheumatoid arthritis; those with neurological diseases such as stroke, cervical myelopathy, and radiculopathy; and those with psychiatric diseases were excluded from both groups because these diseases affect hand movement. All the participants were right hand dominant; therefore, they manipulated the stylus with their right hand. The participants sat on a 40 cm high chair and placed the tablet on a 70 cm high desk. While drawing, the elbows and ulnar sides of the hands rested on the desk and the tablet. They drew three spirals, and the last of the three was analyzed.

### 2.2. App Design

We developed a tablet app to measure the trajectory and pressure of the stylus tip when drawing a spiral on a tablet screen using a stylus (Figure 1a). A blue guide spiral was drawn on the app screen, and the participants used the stylus to draw another spiral along the guide spiral (Figure 1b). As examples, 3D graphs with the stylus trajectory on the xy-plane and the pressure of the stylus tip on the vertical axis for non-CTS and CTS participants are shown in Figure 1c,d, respectively. The longitudinal diameter of the guide spiral was set to 4 cm to observe their manual dexterity and is expressed by the polar equation,

(1) $$r=-\theta \;\left(0\;\leq \;\theta \;\leq 7\pi \right)$$

where r and θ represent radius and angle, respectively. After measuring the trajectory and pressure of the stylus tip, the data were transferred to Elasticsearch (version 7.4, Elastic, Amsterdam, The Netherlands) deployed on Amazon Web Service.

We used a 1st generation iPad Pro 11″ (Apple, Cupertino, CA, USA) and 2nd generation Apple Pencil (Apple, Cupertino, USA), which were among the most popular tablets and styluses available at the time. The frame rate of the tablet screen was 120 fps, and the range of the pressure of the stylus tip was between 0 and 4.166667, which was the custom unit defined by the tablet’s operating system. To improve the drawing comfort and grip of the Apple Pencil, we attached a Pencil Barrier 2 (JJT, Tokyo, Japan) to the Apple Pencil and a paper-like film (MS factory, Kyoto, Japan) to the screen. The app was developed using Unity (version 2019.2.17f1, Unity Technologies, San Francisco, USA).

### 2.3. CTS Classification Using a Support Vector Machine

We created a two-class classification model, non-CTS and CTS, using a support vector machine (SVM) [19], based on the data obtained from the app we developed. To classify patients into the non-CTS or CTS groups, we prepared four training data points for the SVM (Table 1).

### 2.4. The Jerk of the Trajectory and Pressure of the Stylus Tip 

**Figure 2:**
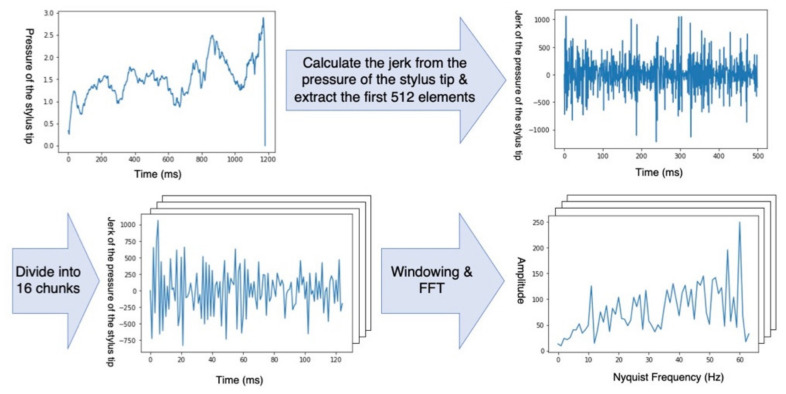
We calculated the jerk from the time-series data of pressure of the stylus tip and extracted the first 512 elements, then applied the Hanning window function and fast Fourier transform (FFT). Finally, the Nyquist frequency, which is half of the sampling frequency, was used as training data.

We preprocessed time-series data of the trajectory and pressure of the stylus tip measured by the app we developed (Figure 2) and used them as training data for the SVM.

**Figure 3:**
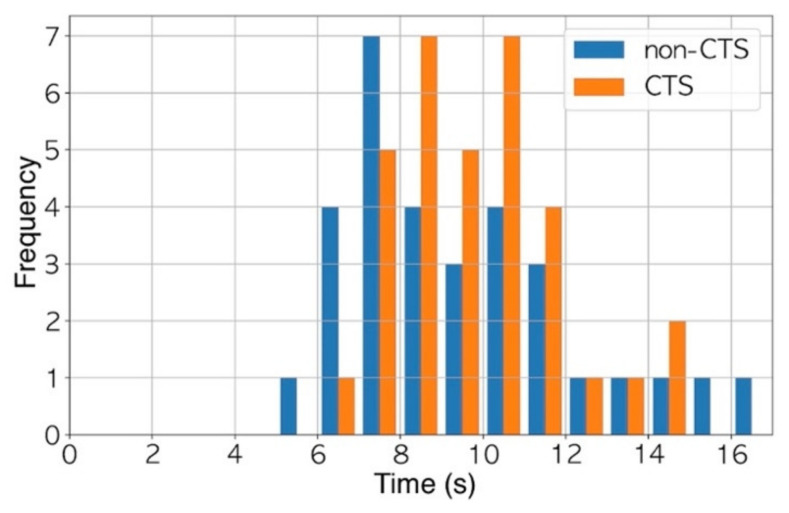
All participants took at least 5 s to complete the drawing.

Firstly, we calculated the jerk from the trajectory and that from the pressure of the stylus tip for each participant. This was because the velocity of the stylus movement and the pressure of the stylus tip differed among the participants. The sampling frequency was 120 Hz. The first 512 frames were extracted from each jerk due to the following two reasons: The first was to enable a classification independent of the time used to draw the spiral—all the participants used more than 5 s to complete the drawing; therefore, we obtained data on a duration less than 5 s for any participants (Figure 3). The second reason was to ensure a quick computation of the fast Fourier transform (FFT) described below when the number of data points is a power of two [20]. We divided the 512 frames into 16 chunks; thus, each chunk consisted of data from 32 frames. Finally, we obtained the frequency components by applying the Hanning window function and FFT to each chunk. Each chunk was converted into 32 frequency components. According to the Nyquist–Shannon sampling theorem, frequency components of up to 60 Hz, which was half of the sampling frequency, were used as the training data. At the end of the training, a 256-dimension dataset (16 chunks x 16 frequency components) was obtained for each participant.

### 2.5. Root Mean Square Error between the Participants’ Spirasl and the Model Spiral

**Figure 4:**
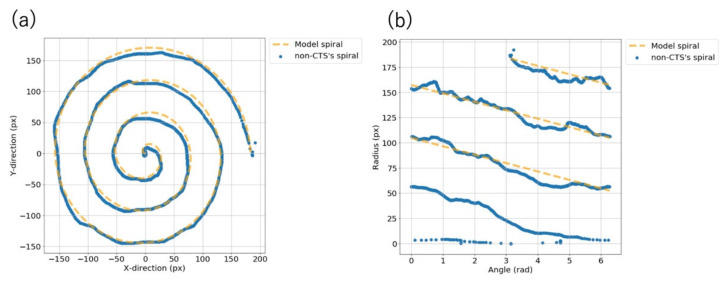
(**a**) Spiral plotted in the Cartesian coordinate system. (**b**) Spiral plotted with θ on the horizontal axis and r on the vertical axis. The solid line is the spiral drawn by a non-CTS participant, and the dashed line is the model spiral. We calculated the root mean square error between the solid and dashed lines.

We calculated the root mean square error (RMSE) between the spirals drawn by the participants and the model spiral. The spiral was not in the Cartesian coordinate system (Figure 4a) but was transformed into a polar coordinate system with θ (0 ≤ θ ≤ 2π) to simplify the observation. We then obtained the spiral plotted with θ on the horizontal axis and r on the vertical axis (Figure 4b) and calculated the RMSE.

The section from the start of the spiral to 2π was excluded from the observation area. This was because when the participants started drawing from a position off the starting point, noise was generated (Figure 4b). We tried to eliminate this noise by using thresholds of θ and r, but this was difficult because the noise generated was different for each participant.

### 2.6. Statistical Analysis

We used a two-tailed Student’s t-test to compare the age in years of the participants and a Chi-square test to compare sex between the non-CTS and CTS groups. The CTS group was divided into patients with grades 1–3 and those with grades 4–6 based on the Bland classification [17], and we compared DASH score, DASH score (writing), pulp pinch strength, grip strength, and disease duration between the patients with grades 1–3 and those with grades 4–6 using the Mann–Whitney U test. The RMSE between the spiral drawn by the participants and the model spiral and the maximum pressure of the stylus tip between the non-CTS and CTS groups were compared using the Mann–Whitney U test. In addition, we compared their data between the patients with grades 1–3 and those with grades 4–6. A *p* value of ≤0.05 was considered a statistically significant difference.

Regarding the SVM, leave-one-out cross-validation was used for classification accuracy verification. The hyperparameters, such as the degree of the kernel function and regularization parameter, for the SVM were tuned so that the cutoff values of the receiver operating characteristic curves were close to the upper left of the graphs.

Our analyses were performed using Python (version 3.7.3, Python Software Foundation) and a machine learning library, scikit-learn (version 0.21.3, scikit-learn developers).

## 3. Results

### 3.1. Participant Characteristics

The characteristics of the participants are summarized in Table 2 and Table 3. There was no significant difference in age and sex between the non-CTS and CTS groups. Between the patients with grades 1–3 and those with grades 4–6, only DASH score (writing) was significantly higher in the patients with grades 4–6; there was no significant difference in the other characteristics.

### 3.2. Root Mean Square Error between the Participants’ Spirals and the Model Spiral

**Figure 5:**
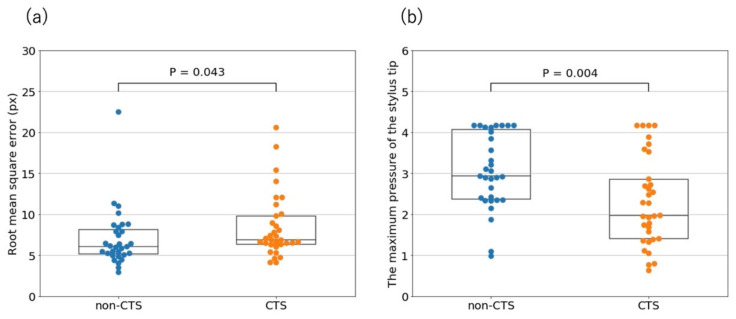
(**a**) Comparison of root mean square errors between the participants’ spirals and the model spiral in the non-CTS and CTS groups. (**b**) Comparison of the maximum pressures of the stylus tip between the non-CTS and CTS groups. There were significant differences between the non-CTS and CTS groups in each comparison.

**Figure 6:**
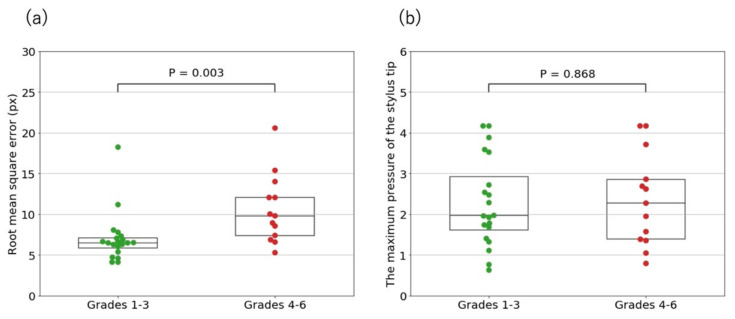
The CTS group was divided into two groups, patients with CTS grades 1–3 and those with CTS grades 4–6, based on the Bland classification. (**a**) Comparison of root mean square errors between the participants’ spirals and the model spiral in the patients with CTS grades 1–3 and those with CTS grades 4–6. There was a significant difference between the grades. (**b**) Comparison of the maximum pressures of the stylus tip between patients with CTS grades 1–3 and those with CTS grades 4–6.

There were significant differences in the RMSE between the participant’s spiral and the model spiral between the non-CTS and CTS groups (Figure 5a) and between patients with CTS grades 1–3 and those with grades 4–6 (Figure 6a).

### 3.3. Maximum Pressure of the Stylus Tip

There were significant differences in the maximum pressure of the stylus tip between the non-CTS and CTS groups (Figure 5b). Contrarily, there was no significant difference in the maximum pressure between patients with CTS grades 1–3 and those with CTS grades 4–6 (Figure 6b).

### 3.4. CTS Classification Using a Support Vector Machine

**Figure 7:**
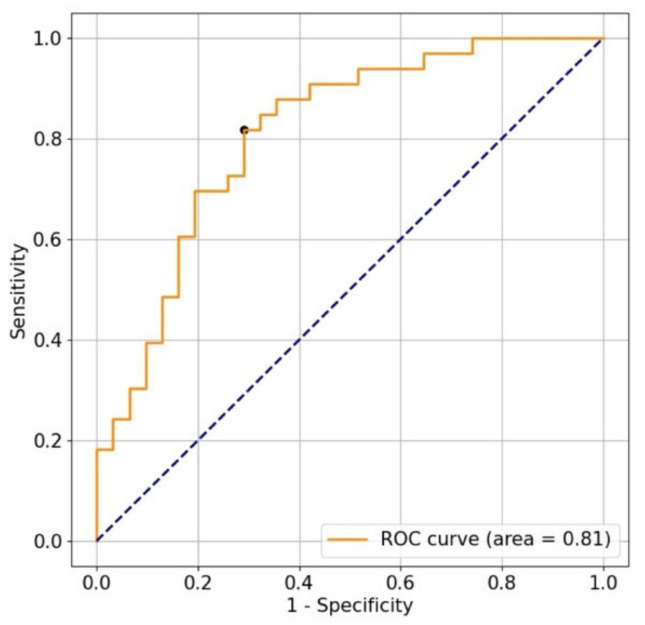
The receiver operating characteristic curve for classification using the jerk of the trajectory of the stylus tip. The sensitivity and specificity were 82% and 71%, respectively, when the cutoff was the black point closest to the upper left of the graph. The area under the curve was 0.81.

The classification using the data from the jerk of the trajectory of the stylus tip as training data showed the highest sensitivity, while the classification using the data from the jerk of the pressure of the stylus tip as training data showed the highest specificity (Table 1 and Table 4 and Figure 7).

## 4. Discussion

In this study, we developed a tablet app that measured the stylus trajectory and pressure of the stylus tip for CTS screening. The sensitivity and specificity of the classification based on the jerk of the stylus tip trajectory were 82% and 71%, respectively (Figure 7). The tablet app in our study did not show high sensitivity compared to that of a previous tablet app [5], which showed 93% sensitivity and 73% specificity, and to that of a previous smartphone app [6], which showed a sensitivity of 94% and specificity of 67%. Contrarily, our specificity was as high as that of the other apps. Previous screening apps [5,6] focused on the failure of the thumb motion caused by atrophy of the thenar muscle. We thought that the apps made it difficult for the patients to perform compensatory movements for the failure of the thumb motion, which was otherwise capable of expressing the characteristics of the failure sufficiently. In our study, in contrast, the flexor tendons of the index and middle fingers compensated for atrophy of the thenar muscle; therefore, it may not have been as well characterized as that of the apps, which was the reason why our sensitivity was not as high as that of the apps. However, as an advantage, our method does not require patients to learn a special game and only involves drawing spirals on a tablet screen, making it easy even for older adults who are not familiar with games. We believe that developing screening methods that require a variety of hand motions can help identify potential patients in the early stages of CTS.

The two sets of training data, the jerk of the trajectory and jerk of the pressure of the stylus tip, showed higher sensitivity and specificity in this study. This implies that our screening can be used if any of the data, the jerk of the trajectory or jerk of the pressure of the stylus tip, are available. Considering that cheap tablets cannot measure the pressure of the stylus tip but can measure the stylus trajectory, our app will help in identifying various patients with CTS because it works on cheap tablets.

Regarding the RMSE between the participants’ spirals and the model spiral, there were significant differences between the non-CTS and CTS groups (Figure 5a), and between the patients with CTS grades 1–3 and those with CTS grades 4–6 (Figure 6a). Compared to the non-CTS group and patients with CTS grades 1–3, patients with CTS grades 4–6 had severe sensory disturbance in the hand and atrophy of the thenar muscle, which seemed to affect manual dexterity, such as accurate drawing. Contrarily, patients with CTS grades 1–3 had mild sensory disturbance and pain in the hand, which seemed not to affect accurate drawing. Additionally, 20 of the patients in the CTS group, comprising the majority, were classified as having CTS grades 1–3, which may explain why the RMSE did not contribute to the CTS classification.

A previous study [9] examined manual dexterity by comparing the maximum pressure of the stylus tip between the non-CTS and CTS groups and reported a significant difference between the groups. Our study also showed similar results (Figure 5b), but the maximum pressure of the stylus tip was higher in the non-CTS group than in the CTS group, which contradicts the observations in the previous study. This could be attributed to the different writing motion used in their study; their study participants drew Arabic numerals, and in this study, the participants drew spirals. Since Arabic numerals are one of the characters that participants are accustomed to writing daily, there is a possibility that individual habits may interfere and may be falsely tagged as abnormal motion. Furthermore, the writing accuracy was not examined in the previous study, even though the participants traced the thinly written numerals with a pen, and it took time to evaluate the ten numerals. Therefore, referring to another previous study [21], we used spirals that would allow us to finish the test in a short time and generate consistent data. In addition, while the previous study [9] did not consider the severity of CTS, we attempted to explore the manual dexterity caused by CTS by classifying the severity. Finally, the previous study aimed to find indices of significant differences, while we aimed to develop a screening method, which is a critical difference between our study and efforts by others.

Additionally, there was no significant difference between the patients with CTS grades 1–3 and those with CTS grades 4–6 (Figure 6b), which meant severe sensory disturbance and atrophy of the thenar muscle did not affect the maximum pressure of the stylus tip. To maintain the pressure, the flexor tendons of the index and middle fingers may have compensated for atrophy of the thenar muscle [9]. Furthermore, forearm muscles may also have to do with the compensation.

Overall, our analyses implied that severe sensory disturbance and atrophy of the thenar muscle affected accurate drawing, but not pressure of the stylus tip. 

This study has some limitations. It is difficult to use this screening method if users develop CTS in their non-dominant hands, since the non-dominant hand cannot draw as precisely as the dominant hand. Furthermore, this study only examined CTS and did not examine other diseases, such as cerebral infarction, cervical spondylosis, diabetic neuropathy, and cubital tunnel syndrome, which also cause a lack of manual dexterity. In addition, non-disease status, such as muscle weakness based on aging and non-painful joint deformity, may also affect manual dexterity; therefore, we will attempt to compare the findings before and after intervention (pre- and post-carpal tunnel release) to control for the confounding effects of these symptoms, making our method more reliable. This study examined this method of CTS screening by analyzing the spirals drawn by participants. In a future study, we will consider comparing the screening accuracy of other shapes, such as square and sine waves, and incorporate additional parameters, such as altitude–azimuth of styluses, into training data to improve the accuracy. Our aim is to provide a screening method for CTS with movements that fit our daily lives, such as writing one’s name.

## 5. Conclusions

We developed a new tablet app, focusing on drawing motion, for CTS screening that measures the trajectory and pressure of the stylus tip when drawing a spiral with the stylus. Our method uses off-the-shelf tablets, which makes it easier to identify potential patients with CTS and enables quantitative assessment of CTS.

## Figures and Tables

**Table 1:** Prepared training data for the support vector machine.

Index	Training Data	Dimension
①	Jerk of the pressure of the stylus tip	256
②	Jerk of the trajectory of the stylus tip	256
③	RMSE ^1^ between the participant’s spiral and the model spiral	1
④	Maximum pressure of the stylus tip	1

^1^ RMSE: root mean square error

**Table 2:** Characteristics of participants in the CTS and non-CTS groups.

Participant Characteristics		Non-CTS ^1^ Group	CTS Group	*p* Value
Number of participants, N		31	33	N/A ^2^
Sex (female), *n* (%)		18 (58.1)	26 (78.8)	0.074
Age in years, median (IQR ^3^)		64 (55–72)	67 (60–73)	0.280
Bland classification				
	Grade 1	N/A	1	
	Grade 2	N/A	4	
	Grade 3	N/A	15	
	Grade 4	N/A	1	
	Grade 5	N/A	10	
	Grade 6	N/A	2	

^1^ CTS: carpal tunnel syndrome. ^2^ N/A: not applicable. ^3^ IQR: interquartile range.

**Table 3:** Characteristics of participants in the CTS group, CTS grades 1–3, and grades 4–6.

Participant Characteristics	CTS ^1^ Group	Grades 1–3	Grades 4–6	*p* Value
DASH score, mean (SD ^2^)	26.6 (19.4)	24.4 (22.2)	29.7 (13.9)	0.300
DASH score (writing), mean (SD)	1.8 (1.0)	1.4 (0.8)	2.3 (1.0)	0.005
Pulp pinch strength (kg), mean (SD)	2.6 (1.5)	3.0 (1.6)	2.1 (1.0)	0.082
Grip strength (kg), mean (SD)	17.3 (8.8)	18.1 (9.7)	16.2 (7.2)	0.807
Disease duration (year), mean (SD)	2.6 (2.6)	2.7 (2.6)	2.4 (2.6)	0.797

^1^ CTS: carpal tunnel syndrome. ^2^ SD: standard deviation

**Table 4:** Classification results of indices of training data described in Table 1.

Training Data	Sensitivity, %	Specificity, %	Accuracy, %	AUC ^1^
①	76	77	77	0.77
②	82	71	77	0.81
③	61	65	63	0.58
④	73	65	69	0.58
① + ②	73	81	77	0.79
① + ③	76	77	77	0.77
① + ④	76	77	77	0.77
② + ③	82	71	77	0.81
② + ④	82	71	77	0.81
③ + ④	58	84	70	0.70
① + ② + ③	73	81	77	0.79
① + ② + ④	73	81	77	0.79
① + ③ + ④	76	77	77	0.78
② + ③ + ④	82	71	77	0.81
① + ② + ③ + ④	73	81	77	0.79

^1^ AUC: area under curve

## Data Availability

The data generated in this study are available from the corresponding author on reasonable request.
